# 8DEstablishment and validation of a hypoxia-related signature predicting prognosis in hepatocellular carcinoma

**DOI:** 10.1186/s12876-021-02057-0

**Published:** 2021-12-12

**Authors:** Congbo Cai, Lei Yang, Kena Zhou

**Affiliations:** 1 Emergency Department of Yinzhou No.2 Hospital, Ningbo, 315000 Zhejiang China; 2 Gastroenterology Department of Ningbo No. 9 Hospital, Ningbo, 315000 Zhejiang China

**Keywords:** Hypoxia, Hepatocellular carcinoma, GSEA, Tumor microenvironment, Risk model, Immune response

## Abstract

**Background:**

Hypoxia plays a crucial role in immunotherapy of hepatocellular carcinoma (HCC) by changing the tumor microenvironment. Until now the association between hypoxia genes and prognosis of HCC remains obscure. We attempt to construct a hypoxia model to predict the prognosis in HCC.

**Results:**

We screened out 3 hypoxia genes (ENO1, UGP2, TPI1) to make the model, which can predict prognosis in HCC. And this model emerges as an independent prognostic factor for HCC. A Nomogram was drawn to evaluate the overall survival in a more accurate way. Furthermore, immune infiltration state and immunosuppressive microenvironment of the tumor were detected in high-risk patients.

**Conclusion:**

We establish and validate a risk prognostic model developed by 3 hypoxia genes, which could effectively evaluate the prognosis of HCC patients. This prognostic model can be used as a guidance for hypoxia modification in HCC patients undergoing immunotherapy.

**Supplementary Information:**

The online version contains supplementary material available at 10.1186/s12876-021-02057-0.

## Background

Liver cancer is a common malignant tumor with poor prognosis and high mortality, ranking the fourth in tumor death [1]. HCC accounts for 75–85% in liver cancer and is the leading cause of death [2]. Although continuous improvement has been made in HCC therapy, the 5-year survival rate of HCC patients is still less than 20% [3, 4]. As is known to all, OS of HCC patients is closely related to the tumor stage. And it is conducive to effective communication between clinicians and patients if the prognosis of individual HCC could be evaluated in an accurate and convenient way.

Tumor growth affects the surrounding microenvironment, and the tumor microenvironment (TME) promotes tumor progression [5]. Substantial data suggest that hypoxia is an important manifestation of TME [6, 7]. HCC has a high level of vascularization, and lots of oxygen is consumed when tumor grows rapidly. Therefore, TME in HCC seems to be in a statement of low oxygen due to insufficient oxygen supply [8]. Previous study has demonstrated that HCC is the most serious tumor of hypoxia [9]. Abundant evidence is showing that hypoxia can induce an increase in tumor malignancy, including tumor progression, invasion and metastasis, contributing to poor prognosis in HCC [10, 11]. Nowadays immune checkpoint inhibitors (ICI) have become an effective immunotherapy and are popularly used in the treatment of HCC [12, 13]. Meanwhile increasing literature indicated that hypoxic microenvironment would decrease the treatment sensitivity in ICI therapy [7]. And improving the hypoxic microenvironment exhibited a more efficient effect on ICI immunotherapy [14]. Therefore, we put forward that hypoxia-related genes could be used to predict the prognosis of HCC and help the choice of hypoxia ameliorating agents during ICI immunotherapy. Currently there is a lack of hypoxia prognostic models in HCC.

In this study, we developed and validated a prognostic model composed of hypoxia-related genes, which can predict the OS of HCC patients. And it is hopefully to be applied as a potential reference for the use of hypoxia ameliorant during immunotherapy in HCC, so as to improve the prognosis in HCC.

## Results

### Clinical characteristics of the enrolled participants

The RNA-Seq expression and corresponding clinical information of 232 HCC patients were downloaded from the International Cancer Genome Consortium (ICGC) database. Analogously, characteristics of 371 HCC patients were obtained from the Cancer Genome Atlas (TCGA) database. Details about the information of training and validation groups are exhibited in Table 1.

**Table 1:** Summary clinical characteristic of HCC patients

Characteristics	Training group (ICGC N = 232)	Test group (TCGA N = 371)
Age category		
< 65/≥ 65/NA	83/149/0	221/149/1
Gender		
Male/Female	171/61	250/121
Vital status		
Alive/dead	189/43	241/130
Grade		
G1/G2/G3/G4/NA	NA	55/177/122/12/5
Tumor stage		
I/II/III/IV/NA	36/106/71/19/0	171/86/85/5/24
T stage		
T1/T2/T3/T4/NA	NA	181/94/80/13/3
M stage		
M0/M1/MX	NA	266/4/101
N stage		
N0/N1/NA	NA	252/4/115

NA: Clinical data are unknown

### Establishment of the hypoxia risk prognostic model

**Fig. 1:**
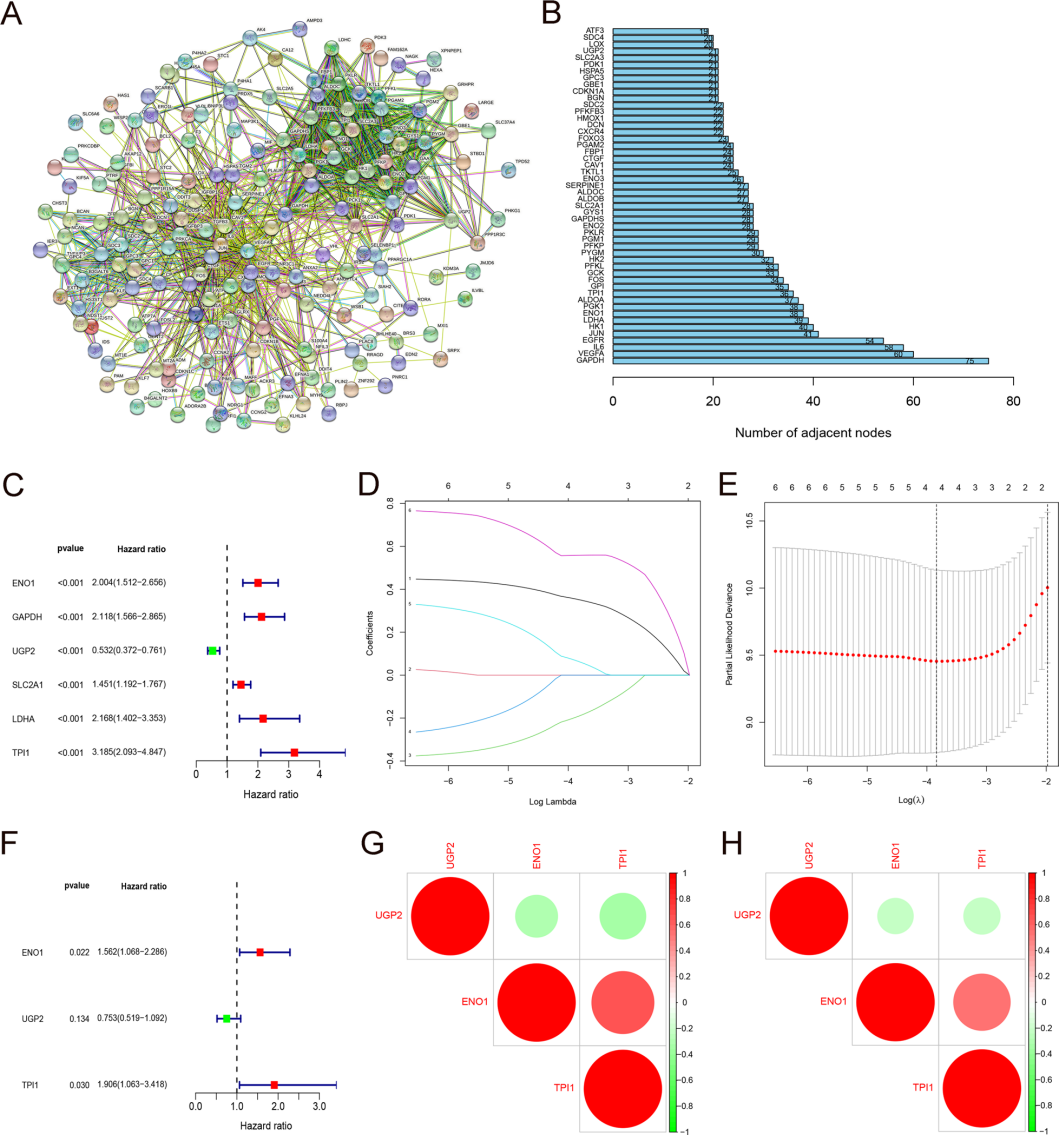
Prognostic model of hypoxia related genes. **A** Protein–Protein Interaction network of 200 hypoxia-relevant genes. **B** Top 50 genes that are most related to each other in PPI network. **C** 6 hypoxia genes closely related to OS in HCC by univariate cox regression. **D**, **E** Establishment of 4-gene model via LASSO regression and lamda value. **F** Forest maps indicating that *ENO1* and *TPI1* are risk genes, while *UGP2* is a protective gene. **G**, **H** Spearman correlation analysis of 3 hypoxia genes in the ICGC and TCGA databases

The genome related to hypoxia was acquired from the gene set enrichment analysis (GSEA) website. These 200 hypoxia-related genes are widely accepted to make research in various clinical studies (See Additional file 1: Data S1 for details). We used the STRING online database (http://string-db.org) to construct a protein–protein interaction network (PPI) for those 200 hypoxia genes (Fig. 1A). The top 50 genes with higher degree of interaction were preliminarily identified to play an important role in the hypoxia process of HCC (Fig. 1B). Univariate cox regression analysis determined that 8 hypoxia-related genes were significantly related to the OS of HCC patients in the ICGC training group (*P* < 0.001) (Fig. 1C). These 8 prognostic-related genes were further analyzed in Least Absolute Shrinkage and Selection operator (LASSO) regression to establish a hypoxic prognosis model for HCC patients. Ultimately, three genes (ENO1, UGP2, TPI1) were selected for the construction of hypoxia prognostic model according to the lamda value in LASSO regression. Among them, ENO1 and TPI1 are risk genes, and UGP2 is a protective gene (Fig. 1D–F). The three prognostic genes in the training and verification groups are closely correlated with each other, positive relation in red and negative in green (Fig. 1G, H).

### Validation of the hypoxia prognostic model

**Fig. 2:**
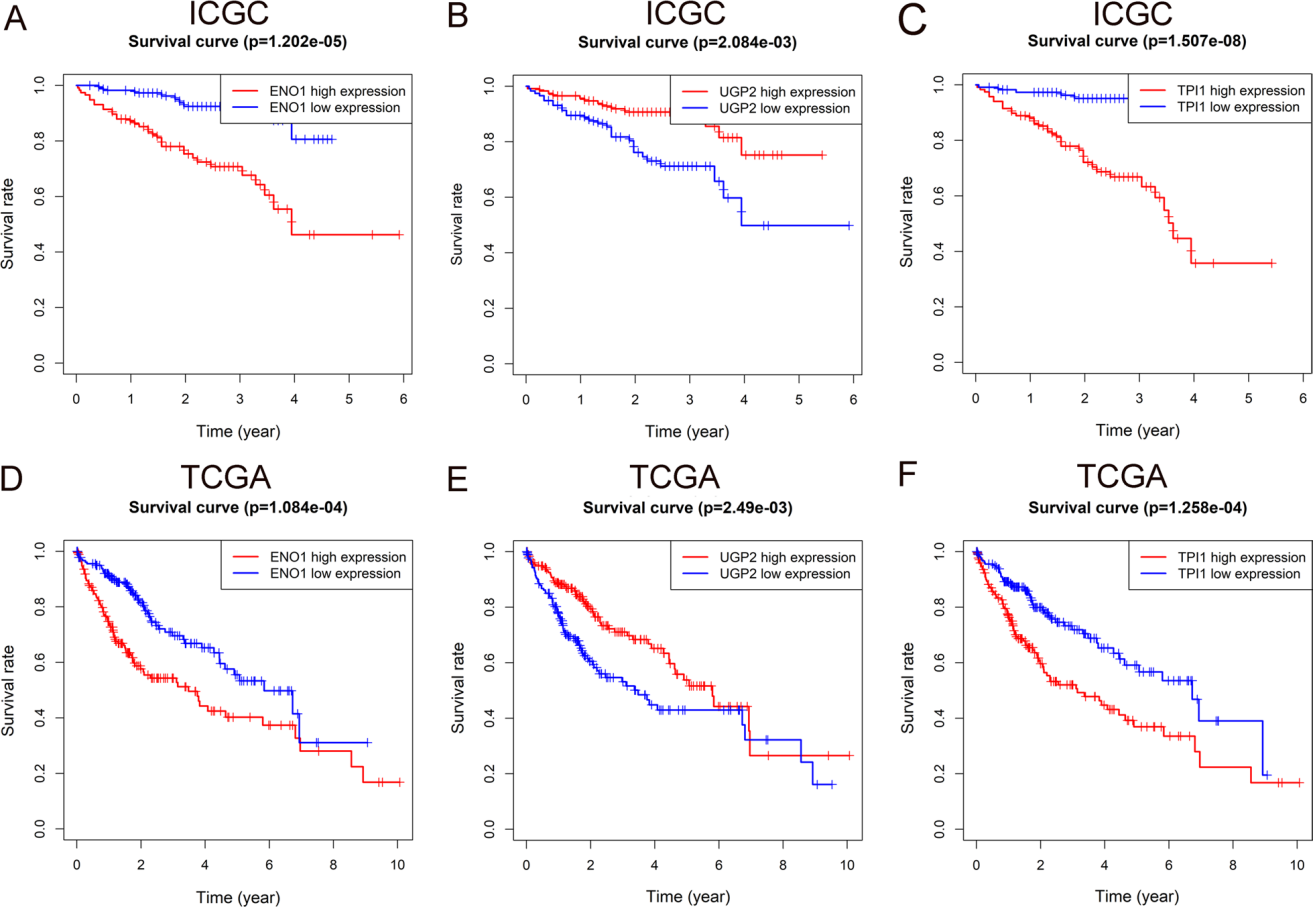
Survival analysis of hypoxia genes. **A**–**C** K–M curves for patients’ survival of *ENO1*, *UGP2* and *TPI1* in ICGC. **D**–**F** K–M curves for patients’ survival of *ENO1*, *UGP2* and *TPI1* in TCGA

We use the Kaplan–Meier (K–M) curve and the risk curve to further validate the hypoxia prognosis model. K–M curves of ENO1, UGP2 and TPI1 in the ICGC queue were drawn for concise analysis. The survival rate of ENO1 and TPI1 high expression group decreased (*P* < 0.01), suggesting that they are risk genes (Fig. 2A, C). On the contrary, the survival rate of the UGP2 high expression group increased (*P* < 0.01), indicating that UGP2 is a protective gene (Fig. 2B). Likewise, similar results were confirmed in TCGA (Fig. 2D–F).

**Fig. 3:**
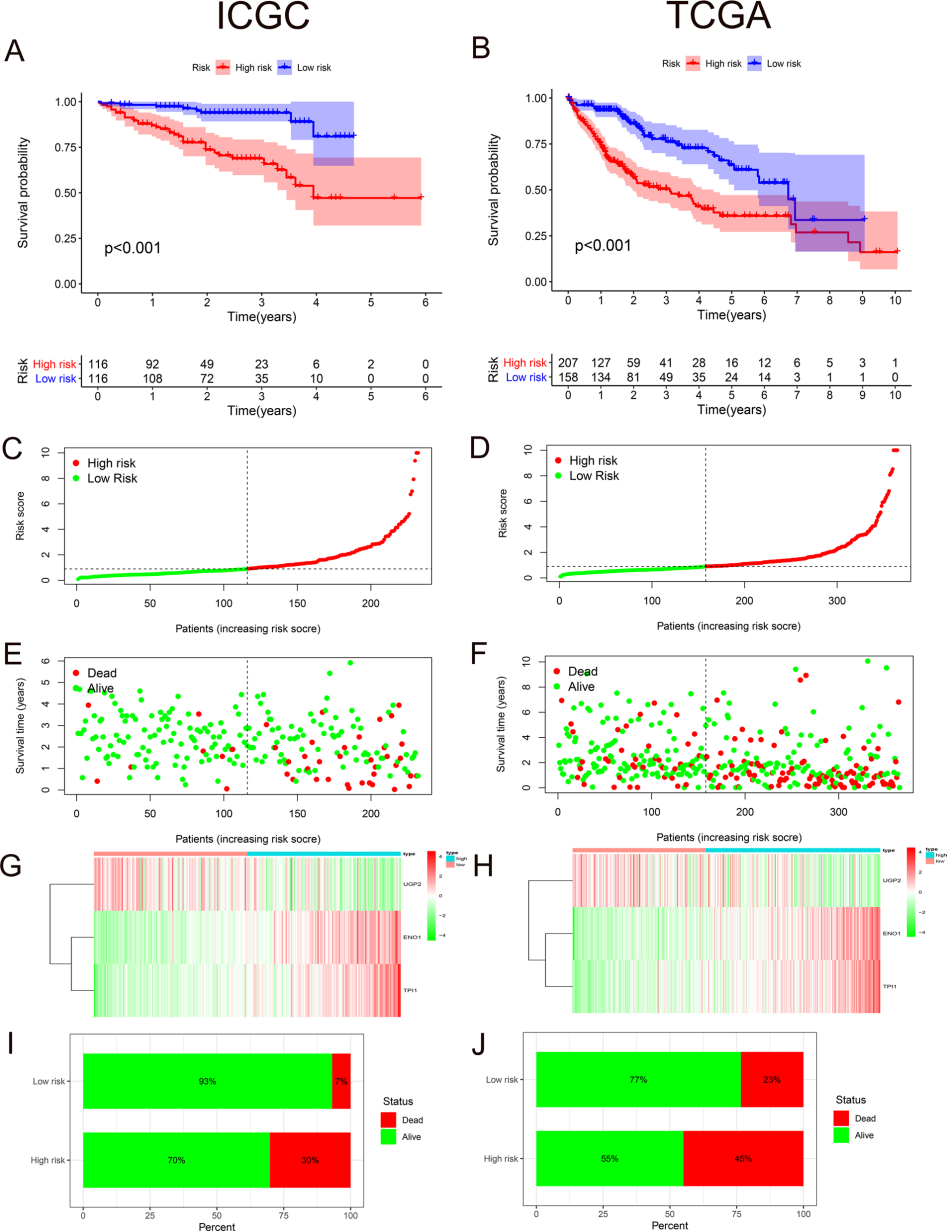
Prognostic value and external validation of hypoxia genes. **A** K–M curves of patients in high- (red) and low-risk groups (blue) of ICGC. **B** K–M curves of patients in high- (red) and low-risk groups (blue) of TCGA. **C**, **D** Sorting patients according to risk score in ICGC and TCGA respectively. **E**, **F** Association between survival time and risk score in ICGC and TCGA separately. **G**, **H** Relationship between risk score and the expression of *ENO1*, *TPI1* and *UGP2*. **I**, **J** The mortality rate in high- and low-risk groups of ICGC and TCGA

All specimens are divided into high- and low-risk groups on the basis of the median risk score as a cutoff. The K–M curve in the ICGC cohort showed that the survival rate of patients in the high-risk group was significantly lower than that in low-risk group (*P* < 0.01) (Fig. 3A). And same conclusion was obtained in TCGA validation cohort (*P* < 0.01) (Fig. 3B). Since the risk curve can further verify the reliability of the model, we sorted all patients in the training group in sequence according to risk score (Fig. 3C). We found that the higher risk score is, the shorter survival time a patient has, and the more deaths caused (Fig. 3E). And the expressions of ENO1 and TPI1 increased as the risk score rose, while the expression of UGP2 decreased (Fig. 3G). Definitely we got the same phenomenon in TCGA validation groups (Fig. 3D, F, H). The mortality rate in high-risk group was significantly higher than that in low-risk group (30% vs. 7%) (Fig. 3I), which is also verified by external cohort (45% vs. 23%) (Fig. 3J).

### Evaluation of the ability of hypoxia prognostic model and the correlation of clinical characteristics

**Fig. 4:**
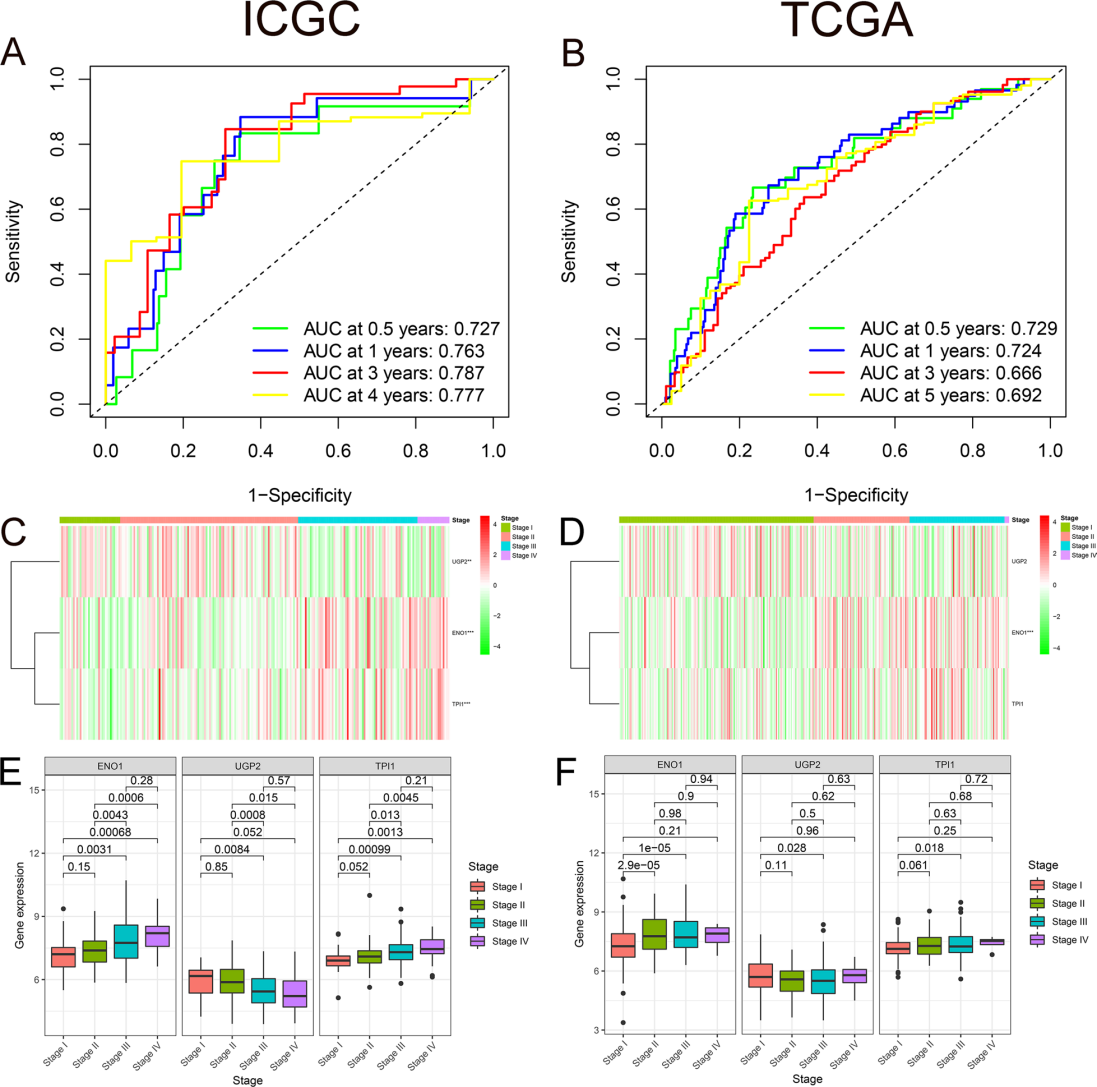
Association between hypoxia and clinical information. **A**, **B** AUC for 0.5-, 1-, 3-, and 4-/5-year OS in ICGC and TCGA. **C**, **D** Heat maps illustrating the association between 3 genes’ expression level and stage stratification in both two databases. **E**, **F** Box plot suggesting the association between 3 genes’ expression level and stage stratification in both two databases

We performed Receiver operating characteristic (ROC) curves on the data from ICGC and TCGA in order to evaluate the prognostic accuracy of the hypoxia prognostic model. And value of area under curve (AUC) for 0.5-, 1-, 3-, and 4-year OS in ICGC were 0.727, 0.763, 0.787, and 0.777 respectively (Fig. 4A). Likewise, the 0.5-, 1-, 3-, and 5-year AUC values in the verification cohort were 0.729, 0.724, 0.666, and 0.692 respectively (Fig. 4B). The concordance index (C-index) value of the model in the experimental group is 0.7551 (95% CI 68.0–83.0%, *P* < 2.0674E−11). The C-index value in the verification group is 0.6802 (95% CI 63.1–73.0%, *P* < 8.6844E−13). The *P* value of the model in the experimental group and the verification group were both < 0.01, indicating that the model has a strong predictive ability. Both the training and validation groups confirmed the good predictive ability of the established prognostic model.

Stage is a traditional clinical tumor stratification with strong practicability in daily clinic. We focused on the relationship between three hypoxia genes and Stage. A heat map was created to explain the association between gene expression level and stage stratification. The expression of ENO1 and TPI1 increased in higher stage levels (*P* < 0.01), while UGP2 expression was weakened (*P* < 0.01) (Fig. 4C). Moreover, quantity analysis was made to identify the relationship between the three hypoxic prognostic genes and the stage level. As the stage level goes up, the expressions of ENO1 and TPI1 increase, and the expression of UGP2 decreases (*P* < 0.05) (Fig. 4E). The expression of ENO1 in the validation group was the same as that in the experimental group. And the expression of UGP2 and TPI1 has no obvious relationship with stage (Fig. 4D, F).

### The independent role of hypoxia prognostic model and the predictive nomogram

**Fig. 5:**
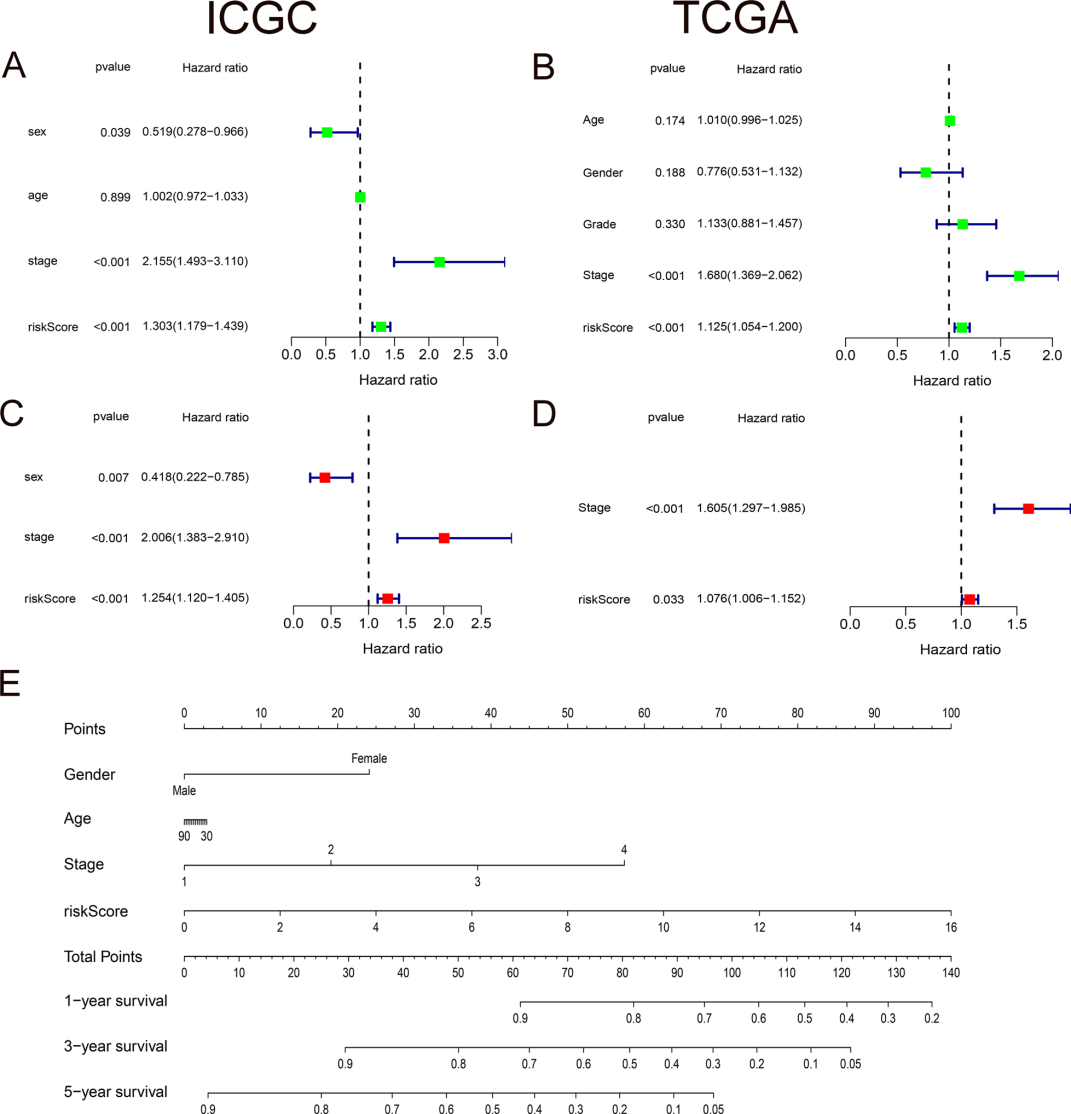
Prognostic value of hypoxia related genes and nomogram. **A**–**D** Independence tests between risk score and clinical characteristics both in ICGC and TCGA. **E** A nomogram predicting the OS in HCC

We also assessed whether the hypoxia prognostic model is independent from other traditional clinical features. The results showed that stage stratification and risk score were independent prognostic factors of OS in the ICGC cohort, both with *P* values < 0.01 (Fig. 5A, C). And the same finding is suggested in TCGA validation group (Fig. 5B, D). In addition, we produced a nomogram for HCC patients, which can be used to quantitatively assess the survival time of each individual. The contents included in the nomogram are clinical features such as gender, age, stage, as well as risk score. Every item has a specific score, and the final score is summed up of all factors. A vertical line through the integrated score corresponds to the survival probabilities of 1-, 3-, and 5-years (Fig. 5E).

### Functional analysis of the prognostic model

**Fig. 6:**
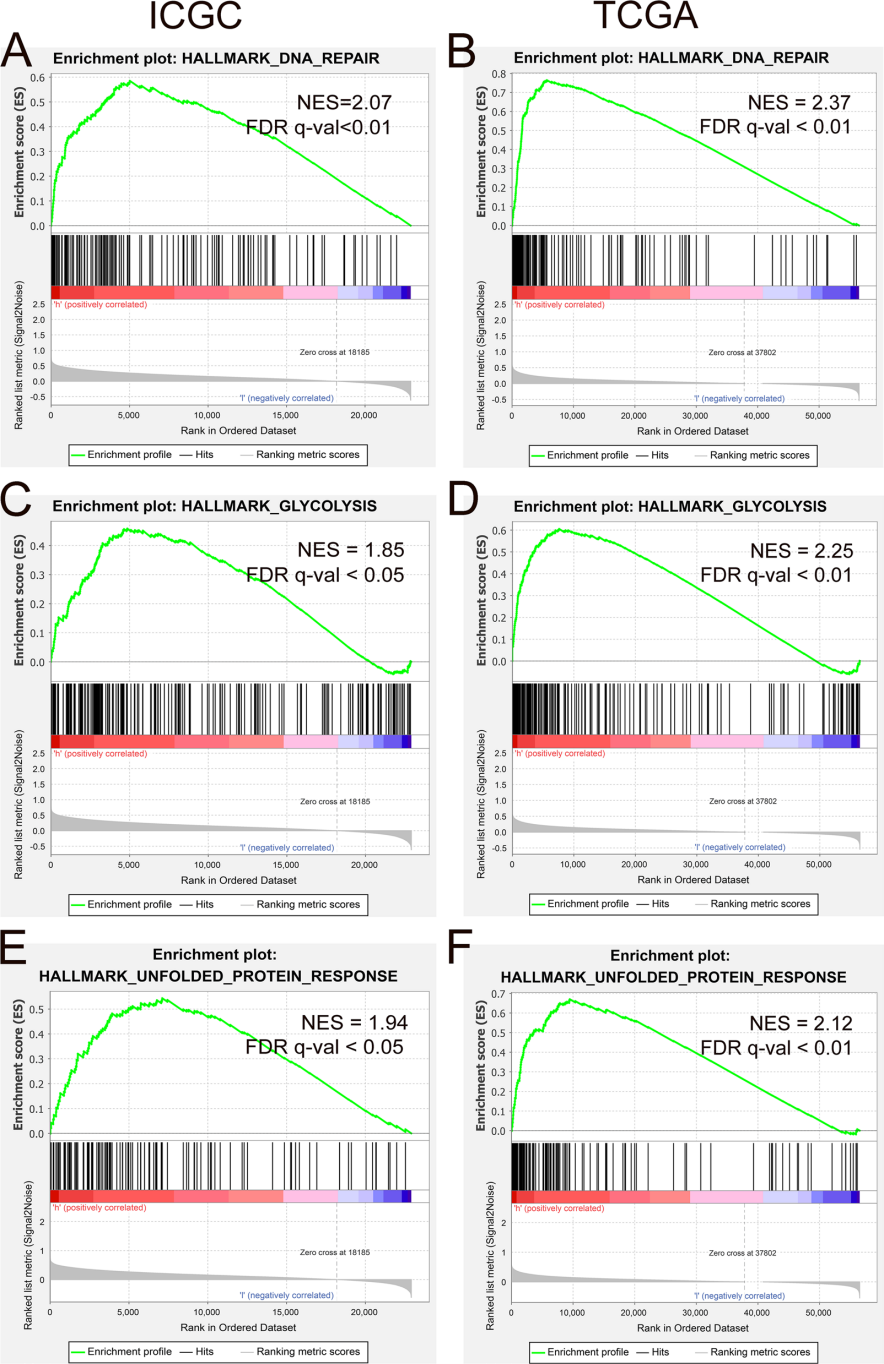
GSEA enrichment of high- and low-risk groups. **A**, **C**, **E** DNA Repair, Glycolysis, and Unfolded Protein responses were enriched in ICGC. **B**, **D**, **F** DNA Repair, Glycolysis, and Unfolded Protein responses were enriched in TCGA

For the purpose of investigating the possible signal pathways related to hypoxia in the progression of HCC, we use the GSEA method to compare the pathway differences between people in high- and low-hypoxia situations. GSEA results told us that DNA Repair, Glycolysis, and Unfolded Protein responses were enriched in both ICGC and TCGA cohorts (Fig. 6A–F).

### Immune cell infiltration among HCC patients in different risk groups

**Fig. 7:**
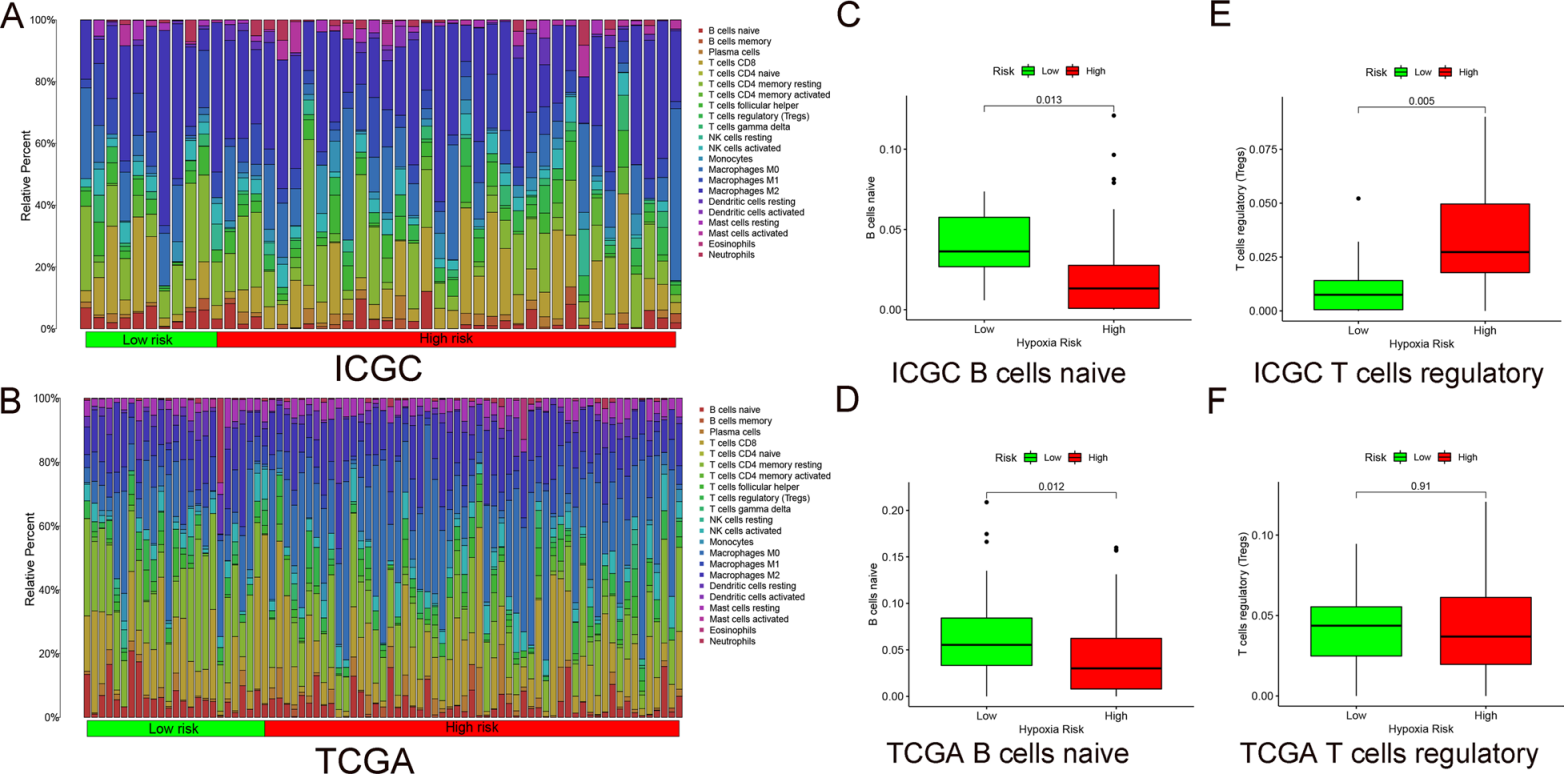
Immune situation in high- and low-risk groups. **A**, **B** Infiltration of 22 immune cells in high- and the low-risk groups of ICGC and TCGA. **C**, **D** B cells naïve in high- and the low-risk groups of ICGC and TCGA. **E**, **F** T cells regulatory in high- and the low-risk groups of ICGC and TCGA

We used the CIBERSORT algorithm to evaluate the degree of immune infiltration of 22 immune cells in the high- and the low-risk groups. A conclusion of ICGC data was drawn in Fig. 7A, and TCGA in Fig. 7B. B cells naive is suppressed in patients with higher levels of hypoxia (Fig. 7C). And the verification results are shown in Fig. 7D. However, the expression of T cells is increased in patients with higher levels of hypoxia (Fig. 7E). And the verification group obtained the same phenomenon (Fig. 7F).

### The immunosuppressive microenvironments and key immune checkpoints in different risk groups

**Fig. 8:**
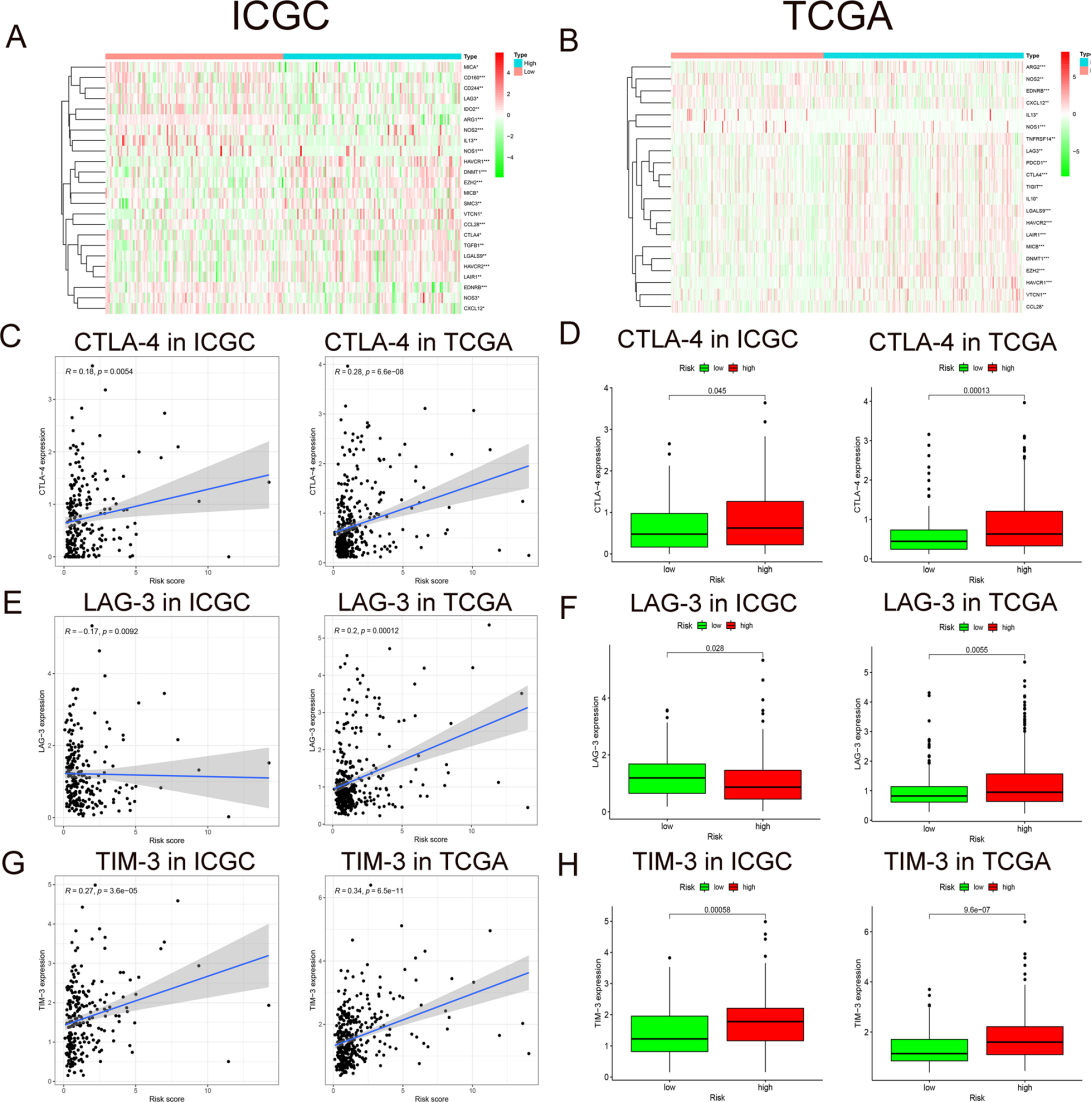
Immune genes in high- and low-risk groups. **A**, **B** Heat maps depicting expression level of negative immune regulatory genes in high- and low-risk groups of HCC from ICGC/TCGA database. **C**, **E**, **G** Correlation between *CTLA-4*/*LAG-3*/*TIM-3* expression level and risk score. **D**, **F**, **H** Box plots of *CTLA-4*/*LAG-3*/*TIM-3* expression level in high- and low-risk groups

Extensive evidence proves that immunotherapy can improve the prognosis of HCC patients significantly [15]. And previous studies suggest that immunosuppressive cytokines play an important role in the TME, especially in the process of tumor development and metastasis [16]. Hence we aim to detect the expression of immunosuppressive genes in high- and low-risk groups of HCC. All immune-related genes were downloaded from the Tracking Tumor Immuno-phenotype website (http://biocc.hrbmu.edu.cn/TIP/index.jsp) (more details in Additional file 2: Data S2). In ICGC cohort, we made a heat map to depict negative immune regulatory genes with differential expression levels (*P* < 0.05) (Fig. 8A). Same graph was produced in TCGA (*P* < 0.05) (Fig. 8B). This demonstrated that many immunosuppressive genes are activated during HCC hypoxia process.

Besides, popular immune checkpoint genes in HCC were also assessed. Our results showed that CTLA-4 and TIM-3 were positively correlated with hypoxia risk score in both ICGC and TCGA (*P* < 0.01) (Fig. 8C, G). And box plots illustrated that CTLA-4 and TIM-3 are up-regulated in the high-risk group (*P* < 0.05) (Fig. 8D, H). However, the immune checkpoint LAG-3 was negatively correlated with the hypoxia risk score in the ICGC cohort, and positively correlated with the hypoxia risk score in the TCGA cohort (*P* < 0.01) (Fig. 8E). Of course same results in box plots of LAG-3 (*P* < 0.05) (Fig. 8F).

## Discussion

The homeostasis of microenvironment is the basis for the stable growth of normal tissues and cells. Predecessors have put forward that the rapid growth of HCC causes a hypoxic TME [17]. Collaboratively, hypoxic TME could promote the deterioration of HCC and increases aggressiveness [18]. Simultaneously, hypoxia can also change the immune status, affecting the prognosis of HCC. Up to now, there is no suitable prognostic model for HCC hypoxia.

In this study, we found that hypoxia has an important effect on the prognosis and immunotherapy of HCC. We developed and verified a model consisted of 3 hypoxia genes (ENO1, UGP2, TPI1), with good prognostic predictive value. Moreover, the nomogram we drew could accurately assess the OS of individual HCC patient, with easier accessibility. And we also found that the hypoxia genes of this model are mainly related to DNA Repair, Glycolysis, and Unfolded Protein response after GSEA enrichment analysis. In addition, we found that the hypoxic TME has an impact on HCC immune infiltration and immune regulation. These discoveries provide a novel insight for predicting the prognosis of HCC patients according to the characteristics of the hypoxic TME. Furthermore, the degree of hypoxia in the TME can be used as a reference indicator for the use of hypoxia ameliorating agents in immunotherapy.

Actually the importance of these hypoxia-related genes has been previously reported in a variety of studies. ENO1 is a key regulator enzyme of glycolysis. Studies have pointed out that the serum ENO1 level in HCC patients was significantly higher than that in normal series [19]. Further studies have shown that the up-regulation of ENO1 promoted cell proliferation, migration and invasion of HCC cells [20]. This is consistent with our research. UGP2 is an essential enzyme for glycogen synthesis. Studies have suggested that UGP2 expression was significantly down-regulated in HCC tissues. And down-regulation of UGP2 expression was equal to poor prognosis in HCC patients [21]. Our research demonstrated that UGP2 is a protective gene of HCC.TPI1 is a key enzyme in the process of sugar metabolism and gluconeogenesis. Previous literature has shown that the enhanced expression of TPI1 in HCC cells in vitro inhibits cell growth, migration and invasion. The survival curve shows that the survival rate of patients is worse when TPI1 is in low expression [22]. However, there are also evidence supporting that OS of HCC patients increased with inactivation of the eight hypoxia genes including TPI1 [23]. And our K–M curve shows that the survival rate of HCC patients increases when TPI1 is low expressed. We believe that these inconsistent results may be due to different environments in vitro and in vivo. Definitely, this requires more basic research to further clarify the function of TPI1 in HCC patients.

GSEA enrichment analysis can effectively explore the possible biological mechanisms of hypoxia prognostic models. Our results indicate that DNA Repair, Glycolysis and Unfolded Protein response are enriched in high-risk group. Substantial evidence showed that hypoxia could cause genome instability and inhibit the ability of DNA damage repair pathways [24–26]. Glycolysis is the main energy source for HCC in hypoxic environment [27, 28]. Cancer cells are often exposed to hypoxia, nutritional deficiencies, oxidative stress and other metabolic disorders, leading to the activation of endoplasmic reticulum stress and Unfolded Protein response [29–31]. Our results indicate that the above pathways are enriched in high-risk patients, revealing that hypoxia is of vital importance in the occurrence and development of HCC.

Tumors can protect themselves from attack by stimulating immune checkpoint targets, such as PD-1, PD-L1, CTLA-4, LAG-3, and TIM-3 [32–35]. Previous literature showed that PD-1/PD-L1 and CTLA-4 are up-regulated at the transcription level under hypoxic conditions, enhancing the immune escape of cancer cells [36–38]. Here we found that the expression of CTLA-4 and TIM-3 in hypoxic patients increased, indicating that the hypoxic TME would promote the immune escape of cancer cells. Many studies have suggested that increasing oxygen in the TME could improve the anti-tumor efficacy of PD-1/PD-L1 and CTLA-4 antibodies [36, 39, 40]. The combination of ICIs and hypoxia ameliorating agents even showed a more effective treatment for HCC patients [41–44]. Thus it is conceivable that the hypoxia risk score has a latent promising value in the use of hypoxia ameliorant during immunotherapy.

In other words, this study has some limitations. First of all, the process of adjusting the weights of regression coefficients in LASSO might ignore some factors that contribute to the prognosis of HCC. Secondly, different standards of inclusion do exist when collecting information retrospectively in different public databases. For example, in stage stratification, ICGC uses the LCSGJ standard, while TCGA uses the AJCC standard. Finally, the complex interaction between tumor cells and immune cells in the hypoxic microenvironment remains to be further explored. More specific experiments are still needed to verify these findings.

## Conclusions

In short, we establish and validated a risk prognostic model developed by 3 hypoxia genes, which could effectively evaluate the prognosis of HCC patients. This prognostic model can be used as a guidance for hypoxia modification in HCC patients undergoing immunotherapy. It will be of great significance to the manipulation of hypoxic stress in comprehensive and innovative immunotherapy in cancer in the near future.

## Methods

### Datasets

The training cohort comes from the ICGC database (https://icgc.org/). The validation cohort is from TCGA database (https://portal.gdc.cancer.gov). Both the ICGC and TCGA databases are freely available to the public, and there is no need for institutional ethics committee approval and informed consent. This research strictly follows the acquisition policy and publication guidelines.

### Constitution of a risk model

Hypoxia-related genes are obtained from the GSEA website (https://www.gsea-msigdb.org/). The 200 hypoxia genes in this group are widely recognized by the academic community for the analysis of tumor hypoxia. The PPI pattern is analyzed in the STRING database (https://www.string-db.org/). Univariate Cox regression analyses was performed to screen out hypoxia genes related to OS (*P* < 0.001). LASSO regression can avoid over-fitting, further optimize the genes selected after univariate cox regression, and delete highly related genes [45]. Finally, multivariate COX regression analysis was carried out step by step to set up a prognostic model. The risk score of HCC patients can be calculated by the following formula: $\mathrm{Risk score}={\sum }_{\mathfrak{i}=1}^{\mathrm{N}}(\mathrm{Ei}*\mathrm{Ci})$, while Ei was the expression value of every three hypoxia genes, and Ci was the corresponding multivariable cox regression coefficient.

### Validation

K–M survival analysis was performed on the prognostic genes in both training and validation groups, in order to assess the impact of a single hypoxia gene on the survival of HCC patients.

Firstly, all risk value of HCC patients in the training group was calculated according to the risk score formula. Then these patients were divided into high- and low-risk groups referring to the median score as a cutoff point. Secondly, K–M survival analysis was carried out to evaluate the predictive ability of the prognostic model. Thirdly, ROC curve is drawn to evaluate the sensitivity and specificity of the prognostic model for predicting survival outcomes. And the AUC would illustrate the accuracy of the prognosis. R package "survcomp" was applied to calculate the concordance index (C-index) of the model to further illustrate the predictive ability of the model. Similarly, the same performance was done in TCGA to evaluate the applicability of the model in an external database. All the above operations are implemented in R language.

### The clinical relevance of the model and production of the nomogram

We performed univariate and multivariate cox regression analysis on the training group as well as the validation group to explore whether the hypoxia prognosis model is independent from other clinical factors. The "rms" software package is used in R language for the construction of the nomogram.

### GSEA

GSEA was utilized to identify groups of related genes that were differentially expressed, further to investigate possible immune mechanisms associated with hypoxia [46]. The GSEA in this study was designed using h.all.v7.2.symbols.gmt. Normally, *P* value < 0.05 and FDR value < 0.05 are considered to witness significant enrichment.

### Analysis on immune cell infiltration

CIBERSORT is the tool most commonly used to analyze immune cell infiltration, which can assess the relative abundance of tumor infiltrating immune cells in different risk groups [47, 48]. In this study, we used CIBERSORT to evaluate the proportion of 22 immune cells in all patients from ICGC and TCGA, and the sum of the scores of all estimated immune cell types is equal to 1. And qualified samples were selected regarding to the standard of *P* < 0.05. Simultaneously, box plots of high- and low-risk groups were drafted in R language.

### Analysis on immune genes

On the purpose of evaluating the expression of immune genes in the high- and low-risk hypoxia groups, we analyze the immunosuppressive genes in the ICGC and TCGA via R language. A heat map of all genes was displayed, and related diagrams and box plots of key immune checkpoints were generated in R language.

## Supplementary Information

**Additional file 1.** 200 hypoxia-related genes were acquired from the gene set enrichment analysis (GSEA) website.

**Additional file 2.** All immune-related genes downloaded from the Tracking Tumor Immuno-phenotype website.

## Data Availability

The datasets used and analyzed during the current study are available from the publicly open databases of ICGC (https://icgc.org/), TCGA (https://portal.gdc.cancer.gov) and GSEA (https://www.gsea-msigdb.org/).

## Abbreviations

HCC: Hepatocellular carcinoma
OS: Overall survival
TME: Tumor microenvironment
ICI: Immune checkpoint inhibitors
ICGC: International Cancer Genome Consortium
TCGA: The Cancer Genome Atlas
GSEA: Gene set enrichment analysis
PPI: Protein–protein interaction network
LASSO: Least Absolute Shrinkage and Selection operator
K–M: Kaplan–Meier
ROC: Receiver operating characteristic
AUC: Area under curve
LCSGJ: Liver Cancer Study Group of Japan
AJCC: American Joint Committee on Cancer
